# The Interrelationship of Reflexivity, Sensitivity and Integrity in Conducting Interviews

**DOI:** 10.3390/bs13030218

**Published:** 2023-03-02

**Authors:** Abdulghani Muthanna, Ahmed Alduais

**Affiliations:** 1 Department of Teacher Education, Norwegian University of Science and Technology, 7491 Trondheim, Norway; 2 Department of Human Sciences (Psychology), University of Verona, 37129 Verona, Italy; 3 Department of English and Communication, The Hong Kong Polytechnic University, Hong Kong SAR, China

**Keywords:** sensitivity, reflexivity, integrity, qualitative research, interview, interviewer

## Abstract

By employing a thematic review of 74 relevant publications and our learning, teaching, and research experiences and expertise, we discussed the concepts of ‘reflexivity’, ‘sensitivity’ and ‘integrity’, and the factors that enhance or hinder their practice. We also categorized the levels of sensitivity according to the stages of conducting and interpreting interviews in qualitative research. By categorizing the three levels of sensitivity ‘i.e., high sensitivity during interviewing, higher sensitivity during transcribing data, and highest sensitivity and criticality during interpreting data’ with practical examples, we offer an approach that facilitates and supports the application of ethical interviews. We conclude that achieving sensitivity and reflexivity enhances the trustworthiness of the overall research and reflects the application of research ethics and integrity in practice.

## 1. Introduction

The rapid increase in the number of qualitative publications in almost all disciplines reflects the trustworthiness and robustness of the research methodology on the one hand, and the need for strengthening specific skills on the other. Among others, the focus is on critical thinking, interview construction and conduct, interaction, data analysis, synthesis, and interpretational skills.

In qualitative research, researchers develop interview guides that help collect in-depth data from research participants. In the conduct of interviews, researchers attempt to exercise empathy, transparency, and unconditional positivity to create an interviewing space [1,2] and interpersonal connection that allows them to establish a good rapport with participants [3]. However, there are moments when either interviewers or interviewees feel that something is not going well in their interaction. This might be attributed to the topic under discussion, which can be sensitive and demands many reflexive experiences on the part of interviewees. This, of course, demands the expertise of inquirers when dealing with sensitive topics.

The previous literature has attempted to differentiate between several types of reflexivity, including the interaction of the researcher with the social world, the sociology of knowledge, publishing and research politics, and using subjectivity to understand the social and psychological world [4]. Despite the trend toward developing a reflexive research paradigm, particularly in the social sciences [5], these benefits of practicing reflexivity have been critiqued for inflating the significance of reflexivity in research or its role in research as a methodological tool [6]. Given the importance of reflexivity in interviewing, researchers have long criticized the dominance of neo-positivism and romanticism paradigms in interviewing [7], and have advocated for the use of interviews as a means of interaction between the interviewer and the interviewee [8]. When researchers misuse reflexivity to selectively extract what serves their aims and fits their beliefs while ignoring the rest of the transcript, this is a tempting interpretation [9]. This argument was also addressed in another study seeking to demonstrate the difference between verbal interview and a verbatim transcript, and its influence on readers.

These days, there is a need to examine moral accountability and apply more practical ways of analyzing and reporting interviews that include more than just selecting specific phrases to address the researcher’s concerns [10]. In this setting, the researcher advocated for more participatory interview interpretation and presentation [11]. This viewpoint is backed up by initiative calls supporting hermeneutic approaches to conduct more meaningful interviews [12]. One idea to help with this stage is to integrate visual and textual information, allowing the researcher to be more reflexive while conducting, interpreting, and utilizing interview results [13]. Given the long debate over what constitutes reflexivity in interviewing and how reflexivity can increase sensitivity in interviews, what matters most is that researchers remain involved in these existing methodological tools [14], in order to gain a deeper understanding and improve their skills in conducting interviews [15] in real-life situations.

Dealing with participants in real-life situations and collecting an abundant number of words demand qualitative researchers to be reflexive and sensitive. In critical qualitative research, the concept of sensitivity is under-researched. Further, the concept of reflexivity differs from one field to another. We believe that the ethical application of both ‘sensitivity and reflexivity’ demands the practice of ‘integrity’. While there are publications on ‘reflexivity in qualitative studies’, there is a lack of studies on ‘sensitivity in qualitative studies’, interviews in particular, or how ‘integrity’ interrelates to the concepts of ‘sensitivity and reflexivity’. As a result, this paper attempts to answer these research questions: (1) What do ‘reflexivity, sensitivity and integrity’ mean in interviews? and (2) How do they interrelate in conducting interviews? Below we report the research procedures and methods.

## 2. Research Design and Methods

We assume that undergraduates, graduates, postgraduates and even academics could achieve a higher level of quality in qualitative research if they are equipped with the sufficient awareness and understanding of interviewees’ needs, interests, and preferences, that is, sensitivity. Further, we contend that this first step could proceed when they are transparent and reflexive, that is, reflexivity. Not only should they be sensitive and reflexive in interviewing, but they should also believe in research ethics and possess integrity, that is, moral development. Given this, we believe that these three work interactively to achieve quality conduct in interviews. It is significant that researchers need to consider conducting qualitative research that contributes to human development with humane, honest, practical, and professional practices.

The thematic analysis technique is useful for reducing researchers’ biases [16], and provides a systematic and thorough analysis of the examined topic [17]. Therefore, we employed the thematic analysis technique because the study integrates the researchers’ profound academic experiences in qualitative research and teaching undergraduate and graduate students, as well as a critical review of previous studies concerning sensitivity and reflexivity in interviewing.

The reviewed literature included publications (articles, book chapters and books), using the English language. We used several databases to explore relevant studies (ScienceDirect, Sage, Web of Science, and Scopus). The starting date was determined using the oldest available papers relevant to the study, and the ending date was determined using the day of the search. Because reflexivity is applied in all topic areas, all subject areas were considered. We used following terms in our search:

1. Title contains sensitivity in qualitative research
2. OR contains sensitivity in research
3. OR contains reflexivity in research
4. OR contains reflexivity in qualitative research
5. OR contains sensitivity and reflexivity in qualitative research
6. OR contains sensitivity and reflexivity in research
7. OR contains adult moral development

We added ‘adult moral development’ in our search keywords because we indicated that researchers might interview with sensitivity, while exercising reflexivity and contemplating integrity. The search resulted in 263 hits, of which 74 were included for review purposes. The rest were excluded due to their irrelevance to the questions of the study, not mentioning any of these targeted themes, or discussing them from different perspectives or contexts. Our list of references shows both the used and cited ones; those with an asterisk (*) refer to the one not used for review purposes.

In this thematic review, we established ‘trustworthiness’ by following four psychometric concepts: credibility, transferability, dependability and confirmability [18,19,20,21,22,23,24,25]. Table 1 details the following techniques used to enhance credibility, facilitate transferability decisions, audit confirmability, and check dependability.

## 3. Findings

In this section, we discuss the main themes and subthemes. Further, we highlight how interviewing with sensitivity by exercising reflexivity, while considering integrity, are interrelated.

### 3.1. Reflexivity in Interviewing

Reflexivity technically refers to the exercise of transparency in interviewing and “promotes an intuition-informed decision-making process as a means to achieve ethical practice and conduct interviews with sensitivity and proficiency” [26] (p. 747). Because reflexivity reflects the attainment of research ethics and quality, some researchers might practice uncomfortable reflexivity instead [27]. Below, we present a synthesis of reflexivity and how qualitative researchers can be sensitive by exercising reflexivity.

#### 3.1.1. Defining Reflexivity

In lexicography, reflexivity is defined as “the fact of someone being able to examine his or her feelings, reactions, and motives…reasons for acting… and how these influence what he or she does or thinks in a situation” [28]. The technical meaning of this concept is not significantly different from the lexical meaning. In particular, the lexeme ‘reflexive’ originated from the theory of Coordinated Management of Meaning (CMM). Cronen and Pearce as cited in [29] introduced the CMM, which assumes that regulative and constitutive rules control human interaction. These rules interact reflexively to form meaningful human interactions. Building upon this theory, two other reflexivity types are generated: the strange and reflexive loops. While the former indicates a change in meaning, the other indicates stillness in meaning [30]. The same author proposed four questions to achieve effective interventive interviewing: lineal, circular, strategic, and reflexive. These affect the interviewer and interviewee(s): conservative, liberating, constraining, and generative effects. They also have different purposes, including explaining problems, behavior, leading and confrontation, and hypothetical future and perspective questions [29]. Regardless of all this conceptualization, the general meaning of reflexivity in interviewing relates to “reflecting on the speaker’s narrative, expressing the interviewer’s understanding of it” [31] (p. 3).

While reflexivity decreases subjectivity in conducting interviews [32], the use of the ‘reflexivity’ concept remains dissimilar according to the context and field of study: political and forensic science [33], health care and midwifery practice [34], supervisor–supervisee relationship [35], interviewing offenders [36], and indicating truth in the literature [37]. Most interestingly, some researchers in the field of psychology use psychoanalysis to reach the unconscious processes and gain knowledge from interviewing [38]. The concept of reflexivity in interviewing extends to folklore research [39], clinical psychology [40], surgery [41], and erotic reflexivity in sociology, where these emotions make the collected data more informative and productive [42].

#### 3.1.2. A Brief Debate on Reflexivity

The practice of reflexivity is the essence of learning to conduct quality qualitative research [43]. With reflexivity, we understand the value of all the participating members in the research, including the researcher, interviewer, interviewees, society, and the surrounding environment and context [44]. However, some researchers have fashionably used it to claim trustworthiness [45], and the evidence concerning reaching reflexivity is still variable. For instance, some researchers argue that the use of audio and visual aids urges the interviewees to emphasize their identity, enhancing reflexivity [46]. There is also an argument that awareness of the difference between contextual and cognitive interests is the path to producing more reliable knowledge using interviews. This argument attempts to distinguish between the society as a whole and the researchers—making and creating knowledge [47]. Above all, we argue that ‘reflexivity’ is an ongoing part of the research process and is a tool that aids in enhancing the interpretation of the data.

The most debatable issue concerning qualitative research is ‘subjectivity’ [48]. The direct interference, and the interviewer’s influence and interpretation of data might reflect questionable reflexivity; we assume that practicing sensitivity helps bridge this gap by increasing the trustworthiness of the investigated knowledge. However, subjectivity is a plus when merged with the examined object or problem (i.e., objectivity) [49], and when merged with the expertise of the researcher to use the data [50]. Researchers argue that there is a possibility for the occurrence of both subjectivity and objectivity in interviewing; a complete picture of the investigated phenomenon can be better seen through this mixture of being subjective and objective during interviewing, transcribing, and interpreting [51].

#### 3.1.3. Factors Enhancing and/or Hindering Reflexivity

Linking the interviewer, interviewees and the society to build up a social world [52] is a factor for enhancing reflexivity. The elicitation of prospective and retrospective reflections over time [53], writing about the personification while conducting research [54], the embodiment of the experiences of the researcher [55], and the interactions among the interviewer, interviewees, and context [56] are all factors that enhance reflexivity. The interaction between the researcher’s body and speech is also influential during interviewing [57]. Moreover, using different categories of knowledge (for example, experiential, clinical, cultural, and academic) strengthens the interview interaction [58]. Additionally, the bioecological systems theory considers space and time as reflexivity. In other words, the geographic location (macro-level) and the immediate surroundings (micro-level) are two factors that can improve the interview quality. Time, be it past, present, or future, improves the interview process even during the transcription and interpretation processes [36].

It is also possible that other factors hinder reaching reflexivity in interviewing. Such factors relate to, for example, touchy topics (e.g., intimacy) [57], losing the focus of the researched topic while interviewing and/or interpretation [32], and the researcher’s values, beliefs, experiences, and interests [59]. Other factors could be the nature of the topic itself, the level of the risk (e.g., the low-risk issue of rural living, gender, the high-risk case of alcohol use), and the trait of the interviewer (e.g., neutrality, self-disclosure) [60]. Further, the researcher’s positionality, be it static or fixed, insider or outsider, contributes either negatively or positively to the reflexivity of the interviewing process [61].

#### 3.1.4. Levels and Categories of Reflexivity in Interviewing

Several researchers have attempted to create a three-level typology of ‘micro, meso, and macro’ levels, which are applicable at the individual, organizational, and societal levels. The use of these three levels leads to three types of reflexivity: self-critique, objective and methodological, and political or social [62]. Furthermore, there are three levels of talk during interviews: personal, interpersonal, and positional. While the personal level focuses on the participant’s specific, unique experiences and feelings, the interpersonal level considers the use of words, images, or metaphors, and how the interviewer and interviewee jointly construct the narrative. How people position themselves in the subject and what they talk about refers to the positional level [63] (p. 238).

While reflexivity in research methods and designs of interviewing is known as methodological reflexivity [40], we have found several other categories of reflexivity. For example, participant reflexivity, as a significant factor in decreasing the interviewer’s subjectivity [64], helps achieve trustworthiness in interviewing [65]. Additionally, participant reflexivity is more credible when using dialogic interviewing; this is supported by three strategies: probing questions (i.e., seeking deeper insights), participants’ reflections (discussing interviews, transcript, interpretation, and even findings with the participants), and counterfactual prompting (leading the participants towards a different perspective of thinking) [66]. The more the participants are engaged, the more the trustworthiness is reachable [67].

Another category is reflexive pragmatism; it is achieved by the “interplay between research design and research questions, interviewing and written product” through “the relationship between epistemology and method” [68] (p. 610). A further category is analytical reflexivity, which requires a thick description of all the processes and factors motivating the researcher to decide or conclude on the searched topic [69]. Moreover, the form of language, be it direct speech, reported speech, or enacted scenes, is also vital in establishing analytical reflexivity in interviewing [70].

**Figure 1:**
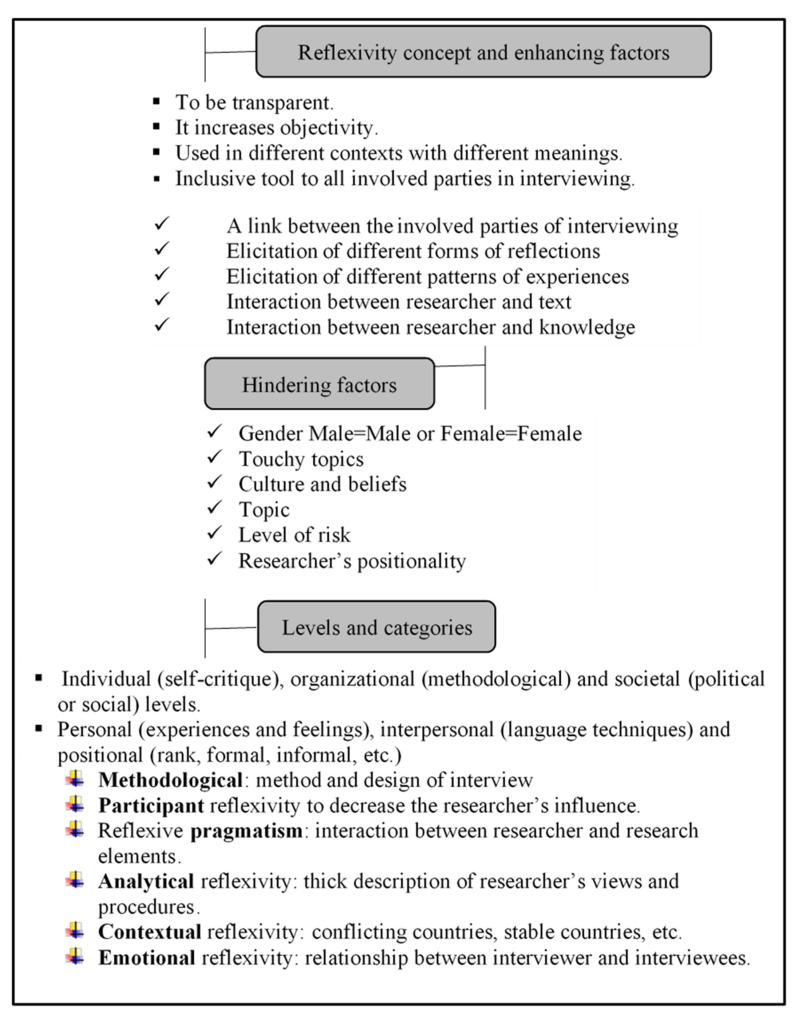
A summary of reflexivity in interviewing.

Researchers in developed and stable countries face different types of challenges while conducting interviews. They, in best cases, view reflexivity as a procedure [71]. Because of the importance of the relationship between the researcher and the participants, and because the feelings of both the parties and the context are vital, emotional reflexivity is introduced as an essential category, referring “to the intersubjective interpretation of one’s own and others’ emotions and how they are enacted” [72] (p. 61). We conclude this part with a summary in Figure 1 of the concept of reflexivity, its enhancing factors, hindering factors, and levels.

### 3.2. Defining Sensitivity: Sensitivity vs. Criticality

The concept of sensitivity is used in medical sciences as a statistical measure for evaluating the accuracy of tests with a positive or negative outcome [73]. The measure of sensitivity becomes highly significant for the reliability of the test outcomes. For medical test results, high sensitivity means high reliability, while the opposite is also true.

In qualitative studies, researchers also exercise sensitivity but in a different way and at various levels. The researcher’s sensitivity includes ‘a host of skills that the qualitative researcher employs throughout all phases of the research cycle’ [74] (p. 780). In interviews, and based on our experiences, ‘sensitivity’ is the concept that deals with awareness and an understanding of interviewees’ needs, interests, and preferences. Being aware and understanding is the primary factor in persuading interviewees to conduct interviews and to engage in possible further interviews and observations. Sensitivity also means that a qualitative researcher needs to be careful in selecting words while interviewing or observing participants so that interviewees are not unintentionally offended. It also means that a researcher needs to be caring, especially when exploring issues that reflect the interviewees’ distressful, depressed, or critical situations. In this sense, sensitivity includes the features of awareness, understanding, carefulness, and caring.

We understand ‘criticality’ as being more relevant to the process of thinking. This critical thinking helps qualitative researchers understand texts, written texts in particular. This means that criticality allows researchers to explore the truth of texts (interview transcripts) while interpreting them. It also means that researchers should be qualified to rationally analyze/interpret and synthesize texts and provide logical conclusions. This criticality is, therefore, very important for all qualitative researchers. In short, sensitivity is applied during verbal interactions with participants, while criticality is implemented in analyzing and reporting texts and raw data.

### 3.3. Levels of Sensitivity While Conducting and Interpreting Interviews

Based on our experiences, we present the three primary levels of sensitivity concerning conducting and interpreting interviews.

#### 3.3.1. High sensitivity: During Interviewing

During the verbal interactions with interviewees, in order to invite them for interviews and during the interviews, qualitative researchers need to have a high level of sensitivity, meaning that interviewers should pay attention to the interviewees’ words, facial expressions, and body gestures, and note them down. It also means that interviewers listen to the (audio-recorded) interactions of the interviewees as a unit, and write some notes regarding information that needs further investigations/questioning.

In in-depth interviews, follow-up questions (probes) derived from the answers of interviewees will occur. High sensitivity in this stage helps the continuity of the interaction in an exciting and rich data-obtaining manner. Showing a high sensitivity while exploring sensitive issues is also critical. It helps make interviewees feel at ease and ready to continue the interaction with trust.

#### 3.3.2. Higher Sensitivity: Transcribing Data

In transcribing data, qualitative researchers must show higher sensitivity in protecting the anonymity of the participants and their interactions. Higher sensitivity should be applied in transcribing all words verbatim without additions or deletions. In the stage of transcribing data, higher sensitivity also includes profiling every interviewee’s interaction separately and with high confidentiality. In this sense, higher sensitivity application consists of the application of research ethics.

#### 3.3.3. Highest Sensitivity and Criticality: Interpreting Data

In interpreting data, qualitative analysts need to have the highest sensitivity level, which indicates the reading of the entire transcripts of interviewees, the natural selection of participants’ quotes and interpreting them without any bias. In this sense, the implementation of criticality is crucial and cannot be separated from sensitivity. It helps conduct a systematic, logical, and reasonable analysis, synthesis, and interpretation of the transcripts/raw data.

#### 3.3.4. Unconscious Development of Hyper-Sensitivity and Its Consequences

Qualitative researchers need to listen to the words of interviewees carefully and pay attention to the interviewees’ tones and intonations, facial expressions, and body gestures (that clearly or partially imply different meanings) for the sake of grasping a complete understanding of the verbal expression. However, they also need to pay further attention to their own words and behavior. This is important because some interviewers might show unhappy feelings (e.g., anger) toward their participants. Although they might not mean it, this shows a negative behavior that affects the flow of interaction, if it does not lead the participants to refuse to continue being interviewed immediately. Showing unhappy feelings during an interaction with interviewees might be attributed to the lack of training the interviewers have received or the presence of hypersensitivity as part of their personality. Both aspects are not favorable for a qualitative interviewer, who thus needs further training on how to employ a moderate or higher level of sensitivity when collecting or interpreting qualitative data. Training on the correct rise and fall of tones/voices is essential to any interviewer and interviewee. This is because such a tone or pitch change in the voice might be interpreted negatively, leading to ending a conversation.

In academic life, researchers interact with one another formally and/or informally. In both formal and informal situations, they need to be very sensitive towards selecting vocabulary and gestures or facial expressions. The arbitrary use of vocabulary, gestures or facial expressions might force others to react hyper-sensitively. Such incidents might put researchers in critical situations when it comes to collecting data through interviews. This is because hypersensitivity might be developed unconsciously.

Giving a first-hand example by the first author here helps understand hypersensitivity and its consequences. Two colleagues respected each other and used to discuss different topics at different times. One is religious, and the other one is not. It happened that they once started a discussion on a religious topic (the presence of God), which should be discussed with caution; here, careful vocabulary should be used and each should show respect to one another’s views.

Scholar 1: … I watched the debate between you and the other scholar on the presence of God. It was interesting …

Scholar 2: Thank you. You see how that scholar was persistent on the idea of the presence of God … which was insane.

Scholar 1: Insane!? Was it!? Why do you think so?

Scholar 2: … hahaha, it seems that you have the same belief …

Scholar 1: And if I have the same belief?

Scholar 2: (Raising their voice with blushing face) you both go to hell!

Scholar 1: Thank you. Bye for now!

While the discussion initially started well, scholar two’s laughter, the raising of the voice, and the utterance of ‘you both go to hell’ indicate that scholar 2 is not aware of the hyper-sensitivity action they have developed for no apparent reason. Although scholar 2 might attempt to exercise sensitivity when conducting interviews, hyper-sensitivity might appear during their conversation with interviewees, as shown in the above example. Scholar 1 ended the discussion respectfully. The consequence is that they have never discussed any topic since then. Indeed, they might not care for each other anymore. Further, and more seriously, the presence of hypersensitivity affects the reliability of collecting and protecting data, and affects one’s academic and personal life.

By considering our own experiences in conducting interviews, we underscore that conducting more interviews helps enhance researchers’ experiences and expertise, and enhances the application of the appropriate level of sensitivity consciously and unconsciously.

### 3.4. Integrity

#### 3.4.1. Sensitivity and Integrity

We attempt to relate sensitivity to adult moral development. This includes discussing the acquisition of sensitivity if we believe that it is acquired just as other elements in our life are (e.g., language). It also consists of the learnability of sensitivity if we assume that this element does not exist within our biological and/or developmental system. By doing so, we attempt to establish how the proposed levels of sensitivity in terms of conducting interviews (i.e., conducting, transcribing, and interpreting) and higher education levels (i.e., undergraduate, master, and (post)doctoral) are possibly acquired, learned, or structured, towards more ethical yet credible qualitative research.

When conducting an interview, moral development plays a vital role during the whole research process. Here, sensitivity in research will be a mixture of knowledge of personal ethics, social rules, and even country policies, regulations, and laws. We quote here the concept of ‘ethical sensitivity’ in order to introduce our concept of “qualitative sensitivity.” While ethical sensitivity refers to “the ability to recognize decision situations with ethical content” [75] (p. 361), our concept of ‘qualitative sensitivity’ indicates a researcher’s capacity to make the participants, readers, and involved society well-informed about how we conduct our research, interview our participants, collect and interpret data, and even inferred or reached our final findings.

#### 3.4.2. Teaching and Learning of Research Ethics

Researchers agree about what could be considered questionable or unquestionable research practices [76,77]. These are usually policies, regulations, or laws, individually or collectively, issued by institutions or countries [78]; this is in order to achieve what we call the ‘academic order’ in knowledge production and science advancement.

Given that there is no concrete evidence regarding whether we acquire ethics or learn them, we believe that this is similar to language, which we acquire during our early childhood and when we move to the preschool level and onwards. Previous and current research on teaching ethics and integrity to students indicates that integrity is an independent variable related to personality, but other external factors could still influence it. For instance, a study on teaching ethics to medical students in Croatia indicates that reasoning relates to gender and Machiavellianism [79]. In the context of Turkey, it is reported that although students realize that it is unlawful to cheat, they still practice it [80]. Nevertheless, viewing sensitivity as ethics based on culture and religion is not enough; rules and laws are vital for implementation. This is evident in countries where higher education quality is low and questionable research practices are practiced [81].

Some researchers tend to practice irresponsible research or questionable research practice [82], including “thinking errors, poor coping with research pressures, and inadequate oversight” [83] (p. 320). However, be it an element of ethics, rules, or laws, the teaching of the responsible conduct of research for a researcher [84] should be promoted regardless of the used teaching methods [85]. These could be case study samples [86], active learning, experiential learning or task-based learning [87]. It could start as early as the undergraduate level and be upgraded based on higher education levels, or be promoted in future careers [88].

### 3.5. The Interrelationship of ‘Sensitivity, Reflexivity, and Integrity’ in Conducting Interviews: Practical Examples

Having introduced each of these three themes separately, we show in Table 2 how these three interact together to allow conducting interviews with higher quality in order to achieve better accountability for qualitative research. We divided the sensitivity levels according to the educational level into three main categories. Next, we provided three possible situations for the three levels of sensitivity when conducting an interview. Each situation shows how a researcher (undergraduate, graduate, or (post)doctorate) interviews, transcribes and interprets data. Criticality, part of sensitivity, is considered during each third level (third, sixth, and ninth). After that, reflexivity is presented, and in each situation, a different category or level of reflexivity is illustrated.

It should be noted that this is changeable according to the situation. This is followed by ‘the typical behavior’ column, which attempts to describe what happened and how it could be modified, benefitting from sensitivity and reflexivity in interviewing. When these two fail, as we illustrated, then moral development (integrity) plays a role. Given this, conducting interviewing requires being sensitive and having intrapersonal and interpersonal skills, being reflexive through transparency and other techniques, and having acquired and learned research ethics. In other words, while moral development is acquiring and learning the knowledge required to conduct interviews ethically, sensitivity and reflexivity are the means and techniques to conduct interviews professionally. It is important to note that the examples provided in the following table are imagined scenarios that help clarify the interrelationship between the ‘sensitivity, reflexivity and integrity’ concepts in conducting interviews.

## 4. Discussion and Conclusions

Our findings critically discuss the high significance of employing ‘reflexivity, sensitivity and integrity’ in conducting interviews and in transcribing and interpreting the collected data. In conducting interviews, reflexivity is useful in increasing the trustworthiness of the collected data. It also helps raise further awareness of the people engaged in the interaction.

Researchers’ reflexivity can be enhanced through eliciting prospective and retrospective reflections over time [53], writing about the personification while conducting research [54], embedding researchers’ experiences [55], allowing interaction between the interviewer, interviewees, and context [56] and using different categories of knowledge [58]. On the contrary, reflexivity can be hindered when discussing sensitive topics (e.g., intimacy) [57], or losing the focus of the researched topic [89], among others. Learning about and practicing the different types of reflexivity is important for interviewers. Methodological reflexivity [40], participant reflexivity [64], reflexive pragmatism [68], analytical reflexivity [69], contextual reflexivity [71] and emotional reflexivity are all important in the ethical and appropriate conduction of interviews. To increase the level of reflexivity, researchers need to also develop some sensitivity.

We argue that the concept of sensitivity deals with interviewers’ awareness and understanding of interviewees’ needs, interests, and preferences. Being aware and understanding is necessary for conducting interviews, in addition to the careful selection of words and showing care while discussing distressing or critical situations. In this sense, we state that sensitivity includes the features of awareness, understanding, carefulness, and care.

The application of our proposed three levels of sensitivity ‘i.e., high sensitivity during interviewing, higher sensitivity during transcribing data, and highest sensitivity and criticality during interpreting data’ are very important and should be learned and exercised by interviewers. By applying these levels of sensitivity, we believe that the ‘qualitative sensitivity’ of researchers is enhanced, leading to a strong application of ‘research integrity’. While teaching and learning research ethics differs from one context to another, it is important that teachers and learners develop a stronger awareness of ‘qualitative sensitivity’, which, if applied well, will lead to the attainment of research ethics and integrity. However, qualitative researchers need to consider not being hypersensitive, as this is not useful in interacting with other people. This demands being careful in our daily interactions, which might shape the way we interact when it comes to conducting interviews.

Finally, we contend that qualitative researchers might apply different levels of sensitivity and reflexivity in conducting and interpreting interviews. The application of a different level of sensitivity and reflexivity, the acquisition of intrapersonal and interpersonal skills, the exercise of reflexive transparency and other techniques, and the learning and teaching of research ethics are significant in conducting and interpreting interviews professionally.

## Figures and Tables

**Table 1:** Techniques used to achieve trustworthiness.

Phase	Concept	Technique	Explanation of the Used Technique
Compiling	Credibility	Peer debriefing	The collected publications were verified and checked by the two authors conversely. The first author reviewed the publications related to reflexivity. The second author checked the publication related to the sensitivity. Titles, abstracts, and/or keywords were used as indicators.
	Transferability	Thick description	A relatively thick description is provided in the methods section for the data collection procedure.
	Dependability	Dependability audit	Auditing was performed between the two authors. The first author examined the data compiled by the second author, who analyzed the data compiled by the first author during the study.
	Confirmability	Reflexivity	The study initially included three investigators but ended with two investigators only. This resulted in dropping the third theme, which was assigned to the third investigator. A discussion between the two authors led to the exclusion of the articles related to this theme.
Disassembling	Credibility	Peer debriefing	The first author proposed some subthemes for presenting data concerning sensitivity in interviewing. The second author reviewed these and suggested some subthemes for reflexivity in interviewing. Again, these were reviewed by the first author.
	Transferability	Thick description	A relatively thick description is provided in this section for the coding procedure.
	Dependability	Dependability audit	The two authors exchanged auditing the key themes and the generated subthemes.
	Confirmability	Reflexivity	Several phone, video calls and chatting between the two authors resulted in the final list of key themes and subthemes.
Reassembling	Credibility	Peer debriefing	The first author matched his generated subthemes to the key theme and the whole study. The second author also matched his generated subthemes to the second key theme and the entire study. The two authors reviewed the key themes (sensitivity and reflexivity) concerning each and the research’s generated subthemes.
	Transferability	Thick description	A thick description is provided in the methods section for generating themes, subthemes and putting them into context.
	Dependability	Dependability audit	The two authors agreed upon the context of each key theme, subtheme and other supporting ideas.
	Confirmability	Reflexivity	The first authors’ initial review resulted in the exclusion of some extracted data due to their indirect link to the study. On the other hand, the themes and subthemes were reordered several times based on reading and the progress of the final version of the study.
Interpreting	Credibility	Peer debriefing	The first author made interpretations for his review for the assigned theme and its subthemes. The second author did the same for his theme and subthemes. The first author reviewed the interpretation and sent it back to the second author for further interpretation and visual illustrations.
	Transferability	Thick description	A thick description is provided in the methods and results sections for the data interpretation procedure.
	Dependability	Dependability audit	The two authors audited the interpretations of each other and the whole work via the raised questions and topic of the study.
	Confirmability	Reflexivity	Verbal discussions between the two authors helped audit and expand the interpretations of the collected data and provide examples and illustrations for answering the questions of the study.
Concluding	Credibility	Peer debriefing	Both authors proposed initial conclusions based on textual and visual interpretations. Both authors checked, verified and decided on the credibility of the proposed conclusions.
	Transferability	Thick description	A thick description is provided in the methods section for the conclusion deduction procedure.
	Dependability	Dependability audit	Both authors proposed conclusions based on their writing experience of the paper and the editing of the whole work.
	Confirmability	Reflexivity	Each author reflected their own experience in studying and working in academia in the findings. Although this was performed as part of the used data to support the study, it was performed carefully and logically in order to avoid any biased views or manipulation of the synthesized collected publications.

**Table 2:** Application of sensitivity levels, reflexivity, and moral development with examples.

Level	A Typical Behavior	Reflexivity	Adjusted Behavior	Moral Development
Moderate for undergraduates Context: An undergraduate student is conducting an interview about research ethics comparing eastern and western standards to apply research ethics. The interviewee is from an Arabian state working as a professor in the same condition. Context: The supervisor calls the student to ask for the recording of the interviews to verify the data collection process.				
High during interviewing	Interviewer: Why do you think research ethics are violated more in developing nations? Interviewee: Talking…. Interviewer: She is looking at the phone and doing other issues to pass the time…	Emotional reflexivity is violated as the researcher fails to establish successful communication. Verbal and non-verbal communication should take place to achieve emotional reflexivity.	The researcher has basic skills, but with the sensitivity tool; the researcher should know that staying connected with the interviewee physically and mentally is vital. This makes more successful communication and encourages the interviewee to produce more honest views.	Following the cognitive development theory and our proposed levels of sensitivity according to the university level, this student is still at the basic level of moral cognitive development. When considering the three situations of interviewing, transcribing, and interpreting, it is typical to see these practices in a young researcher. Further, considering moral development, namely social order, and our proposed concept ‘academic order’, is open to two possibilities. The researcher is still at the basic level of acquiring and learning research ethics. Or the researcher could have already acquired and learnt them but intended to violate them. In that case, it will move to personal integrity.
Higher during transcribing	Interviewer: I recorded using my mobile phone, and the recording was deleted unintentionally. Supervisor: You need to perform your data collection again. Please come to my office to discuss it.	Interaction reflexivity is violated here as the researcher did not care that much about the collected data. The researcher did not take any precautionary steps to avoid the loss of data.	The supervisor had doubts about the collected data and wanted to verify these statements with her supervisee. While the situation is still vague, the transcription was not performed verbatim. In addition, the student seems to have no awareness that the data should be kept for verification.	
Highest during interpreting	Interviewee: Several factors are contributing to this widely spread phenomenon of violating research ethics among our students. However, we should be careful claiming with certainty that we tend to violate research ethics more. Among these factors are education, teaching this subject, economic level, awareness, and honesty. Interviewer: The participant mentioned that students in the developing nations violate research ethics because they are less educated and less honest than other nations who are more educated and honest.	Contextual reflexivity is violated here as the researcher has the initial intention to prove that developing states are negative and prove that other states are positive.	The interpretation of the response provided by the professor is inferior. It is not only poor but also is misinterpreted to match the researcher’s intended meaning. The collected data is fabricated covertly to correspond to the desired message of the researcher. The highest sensitivity was not considered, and could be used to adjust this interpretation, and interpret with honesty and logically.	
Criticality		Criticality applies to the third level of sensitivity here. The student analyzed without critical thinking or any basic analytical skills.		
High for graduates Context: The researcher is an MA student, about to graduate and editing the thesis final draft before submitting to the defense committee. She is politically and economically unstable and contacted a researcher; she used his papers and wanted him to help her check the thesis.				
High during interviewing	Researcher: I went through your extracted data samples from the interviews and noticed that they look so similar, and some of them have a a language level that is too high to look translated from another language. MA student: I know what you mean. To be frank, I did not conduct all the interviews. I do not have enough money and time to do so. I found some answers to some questions from the Internet. I interviewed some people, but I had no time to listen and transcribe everything.	The student violated contextual reflexivity by assuming that being in a country that is not developed is enough to excuse her making up interviews.	The student is at the master’s level and knows that the interview should be conducted in a certain way, but tends to ignore being sensitive, reflexive, and ethical due to financial and personal reasons.	Following the cognitive development theory, the researcher is now more mature and is supposed to have acquired and learnt more typical research ethics and practices. Having a look at these three situations, the researcher seems to be aware of all these issues but lacks personal integrity. Similarly, the researcher is unwilling to achieve neither social order nor academic order. This poor personal integrity is associated with inadequate regulations, laws, and policies in this researcher’s context. Regarding group responsibility in the cognitive development theory, the researcher here is not the only one responsible for such unethical practices.
Higher during transcribing	Researcher: All right. I am telling you that these are not ethical practices in research, especially when you are already a master’s student. Why don’t you just transcribe what you got from the interviews? MA student: I transcribed a few interviews. But I did not like what they said. It does not answer the questions the way I expect or what my supervisor expects. I am afraid they will fail me, so I changed the responses to match my thinking and my supervisor’s thinking.	The societal level of reflexivity was used here to reflect social and cultural elements that impact research ethics and research skills in the society in which this MA student lives.	The students intended to violate research ethics intentionally due to personal reasons. The researcher seems to be aware of being sensitive and reflexive in interviewing but trying to push her situation to excuse unethical practices.	
Highest during interpreting	The researcher: I feel these interpretations are copied and pasted excerpts. I even checked them and found that most of them are marked as plagiarized. MA student: You don’t know what kind of life we have! You live in a country where you have all your needs and more. I am trying to learn. I am just a beginner researcher. I just want to finish my MA and get a job. Who cares if this is mine or someone else’s!	The MA student failed to meet the individual reflexivity and was self-critical of her unethical behaviors and illogical justifications.	This misbehavior seems hard to deal with in terms of sensitivity and reflexivity alone; they are more related to integrity and moral development.	
Criticality		Criticality applies to the third level of sensitivity here. The MA students here think critically, but even think that the research is useless anyway, and she wants to produce her graduate paper to get a job.		
Higher for (post)doctorates Context: The interviewer is completing her doctoral degree in a country A, University A, preparing her final dissertation and publication to graduate. The researcher is an outsider, since she travelled to complete her doctoral degree there.				
High during interviewing	Interviewer: I have finished the interviews. They all gave the same answers. I deleted all the recordings. I no longer need them.	Analytical reflexivity is violated as the researcher fails to describe how it is possible to reach the same views from people who must have different opinions, even if they agree somewhere.	Although the researcher is a doctoral student, her awareness is below the required level for conducting interviews professionally. Sensitivity and reflexivity could help the student to upgrade her interviewing skills.	Now, the doctoral student is at the highest stage of maturity for acquiring and learning research ethics. This is even more the case for postdoctoral academics. Such unethical practices indicate the poor acquisition and learning of research ethics during the first two levels (undergraduate and graduate levels). This is again a group responsibility for why this student reached this level but still had such unethical research practices. At this stage, they intend to disrupt social order and academic order intentionally and systematically. This could be attributed to personal ethics, cultural, social, or even the working environment.
Higher during transcribing	Sam said … . Sarah mentioned … . Bright Kid School principal stated … Interviewer: I told you last time that I already deleted the recording, so I do not remember who said what. Supervisor: You recorded 20 people, and I think we need to have all the data transcribed to analyze the transcripts Interviewer: Sorry, to be frank, I did not delete the recordings. I lost my phone, and all the recordings were stored there. So, I ended up writing answers based on recalling my talk with the participants.	Reflexive pragmatism is violated here as the researcher failed to interact with the research and the researcher successfully. It does not seem that the researcher cares that much for the collected data.	The researcher is careless about the research process. Above all, she is ready to provide false data and do anything to complete the research process. If the student is aware and able to apply the highest sensitivity in interviewing, criticality, and reflexivity, these unethical solutions would have never come to her mind.	
Highest during interpreting	Supervisor: I checked your first draft, but I am afraid I could not see any question in the interview about the cultural reasons for early marriage. However, I remember that you had one question about this issue, and I can see that you provided interpretation for some excerpts. I am afraid that the quote and interpretations are inconsistent.	Participant reflexivity is violated here as the researcher interpreted the data to cover up her mistakes in conducting interviews professionally. The participants’ views are fabricated to cover up the gaps encountered due to poor research skills.	The researcher is a doctoral student but still has poor research skills, including ethical ones. When the student found a gap that was missed, she opted to transcribe subjectively using false interpretation. If the student was competent in interviewing awareness, reflexivity, and integrity, the solutions would be appropriate.	
Criticality		Criticality applies to the third level of sensitivity here. Critical thinking is practiced here but unethically. The student thinks but produces unethical results.		

## Data Availability

Not applicable.
